# Estrogen deficiency heterogeneously affects tissue specific stem cells in mice

**DOI:** 10.1038/srep12861

**Published:** 2015-08-06

**Authors:** Yuriko Kitajima, Hanako Doi, Yusuke Ono, Yoshishige Urata, Shinji Goto, Michio Kitajima, Kiyonori Miura, Tao-Sheng Li, Hideaki Masuzaki

**Affiliations:** 1 Department of Stem Cell Biology, Atomic Bomb Disease Institute, Nagasaki University, 1-12-4 Sakamoto, Nagasaki 852-8523, Japan; 2 Department of Obstetrics and Gynecology, Graduate School of Biomedical Sciences, 1-7-1 Sakamoto, Nagasaki 852-8501, Japan

## Abstract

Postmenopausal disorders are frequently observed in various organs, but their relationship with estrogen deficiency and mechanisms remain unclear. As tissue-specific stem cells have been found to express estrogen receptors, we examined the hypothesis that estrogen deficiency impairs stem cells, which consequently contributes to postmenopausal disorders. Six-week-old C57BL/6 female mice were ovariectomized, following which they received 17β-estradiol replacement or vehicle (control). Sham-operated mice were used as healthy controls. All mice were killed for evaluation 2 months after treatments. Compared with the healthy control, ovariectomy significantly decreased uterine weight, which was partially recovered by 17β-estradiol replacement. Ovariectomy significantly increased the numbers of c-kit-positive hematopoietic stem/progenitor cells in bone marrow, but impaired their capacity to grow mixed cell-type colonies *in vitro*. Estrogen replacement further increased the numbers of c-kit-positive hematopoietic stem/progenitor cells in bone marrow, without significantly affecting colony growth *in vitro*. The number of CD105-positive mesenchymal stem cells in bone marrow also significantly decreased after ovariectomy, but completely recovered following estrogen replacement. Otherwise, neither ovariectomy nor estrogen replacement changed the number of Pax7-positive satellite cells, which are a skeletal muscle-type stem cell. Estrogen deficiency heterogeneously affected tissue-specific stem cells, suggesting a likely and direct relationship with postmenopausal disorders.

The physiological changes caused by urogenital atrophy can induce a number of pathological disorders, such as osteoporosis, thromboembolism, arteriosclerosis, and muscle atrophy, all of which are frequently observed following menopause^1^. These pathological disorders are considered to be associated, at least partially, with ovarian dysfunction, especially estrogen deficiency^2^. Therefore, hormone replacement therapy (HRT) is popularly accepted to prevent/alleviate these postmenopausal disorders^3^. HRT has been found to be beneficial, but is also accompanied by side effects and may induce some complications of its own^4^. As many other common pathogenic factors are also well known to contribute to these disorders, we must confirm their relationship with estrogen deficiency and gain further understanding of the relevant mechanisms at the molecular and cellular levels.

The rapid advancement on stem cell research in past decades has clearly identified stem cells in almost all tissues/organs of our body, and these tissue specific stem/progenitor cells are functionally known to be critical in the repair/regeneration of tissue/organs due to physiological turnover or pathological damage^5^,^6^. Aging and many other pathogenic factors, such as diabetes mellitus, can impair these tissue-specific stem cells^7^. Interestingly, recent studies have found that the resident stem cells in different tissues/organs, including hematopoietic stem cells, endothelial progenitors, mesenchymal stem cells, and satellite cells (skeletal muscle-type stem cells) express estrogen receptors^8^,^9^,^10^,^11^. It has also been reported that estrogen plays critical roles in regulating the survival and differentiation of either embryonic stem cells or tissue-specific stem/progenitor cells^12^. Considering the potential roles of estrogen in maintaining stem cells, it is quite possible that estrogen deficiency causes a decrease in the quantity and quality of stem/progenitor cells, which consequently contributes to postmenopausal disorders in various tissues/organs.

Using an ovariectomy model in young healthy female mice, we herein examined how estrogen deficiency could affect hematopoietic and mesenchymal stem cells in bone marrow, as well as satellite cells in muscles.

## Results

### Estrogen deficiency induced uterine atrophy and increased body weight

**Figure 1:**
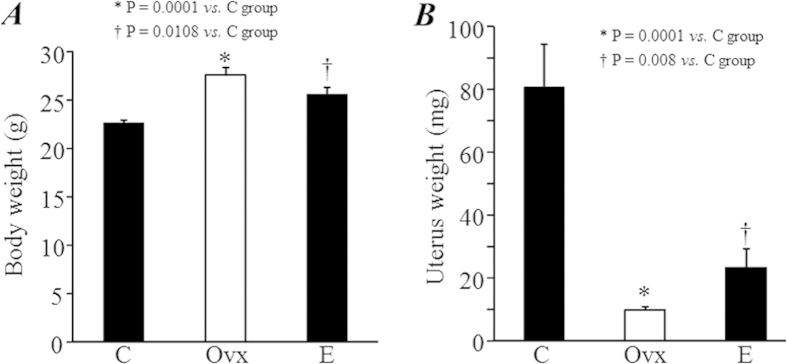
Body weight and uterine weight. Body weight (**A**) and uterine weight (**B**) were measured 2 months after treatments.

The body weight of mice was significantly higher in the Ovx and E groups compared with C group (*P *= 0.0001 and 0.0108, Fig. 1A). The uterine weight was significantly lower in the Ovx group compared with E and C groups (*P *= 0.0001, Fig. 1A), although the uterine weight was also significantly lower in the E group compared with the C group (*P *= 0.008, Fig. 1B). These results indicate that partial atrophy of the uterus occurred following estrogen replacement during the 2 months after ovariectomy.

### Estrogen deficiency increased the number, but impaired the function, of c-kit^+^ hematopoietic stem/progenitor cells

**Figure 2:**
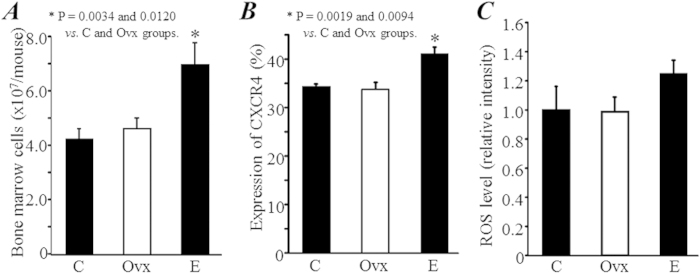
The expression of CXCR4 and intracellular ROS levesl in bone marrow mononuclear cells (BM-MNCs). (**A)** The bone marrow was collected from the femur and tibia 2 months after treatments, and the number of total collected mononuclear cells from each mouse was directly counted. (**B)** The expression of CXCR4 was detected in freshly collected BM-MNCs by flow cytometry. (**C)** The intracellular ROS level was measured as the mean fluorescence intensity in the freshly collected BM-MNCs after 30 min loading with 10-μM CM-H_2_DCFDA.

Although all procedures were performed with well-trained skill and a defined protocol, we collected significantly more BM-MNCs in the E group compared with the C and Ovx groups (*P *= 0.0019 and 0.0094, Fig. 2A). The expression of CXCR4 in BM-MNCs did not differ between the C and Ovx groups, but was significantly enhanced in the E group (*P *= 0.002 and 0.009 *vs.* C and Ovx groups, Fig. 2B). The intracellular ROS level in BM-MNCs was not significantly different among groups, although it was slightly increased in the E group (Fig. 2C).

**Figure 3:**
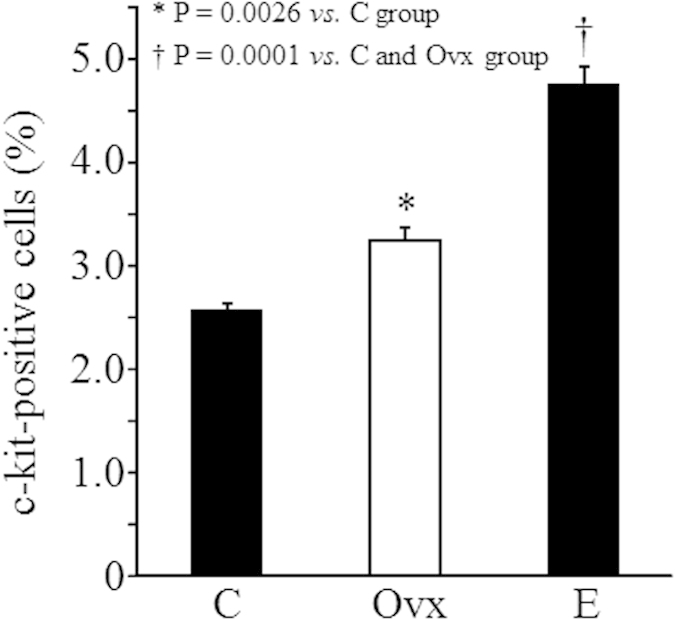
The number of hematopoietic stem/progenitor cells. The expression of c-kit, a marker of hematopoietic stem/progenitor cells in bone marrow mononuclear cells, was measured by flow cytometry.

Moreover, compared with healthy mice in the C group, the expression of c-kit, a marker popularly used for identifying hematopoietic stem/progenitors cells, was detected to be significantly higher in the Ovx group (3.27 ± 0.14% *vs.* 2.65 ± 0.09%, *P *= 0.0026; Fig. 3). Strangely, the expression of c-kit was even further increased by estrogen replacement (4.87 ± 0.16%, *P *= 0.0001 *vs.* C and Ovx groups, Fig. 3).

**Figure 4:**
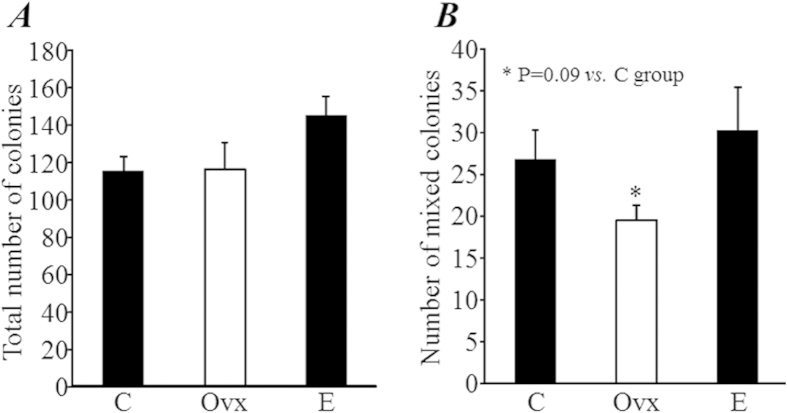
Colony-forming assay. Bone marrow mononuclear cells were isolated from mice 2 months after treatments. Colony formation was observed under microscopy at 7 days after incubation. The number of all types of colonies (30 cells, (**A**)) and mixed cell type colonies (at least two different types of cell in the colony, (**B**)) were counted.

The results of the colony-forming assay, a method well used for evaluating the function of hematopoietic stem/progenitor cells *in vitro*, showed that the total number of colonies formed from 2 × 10^4^ BM-MNCs was not significantly different among groups (Fig. 4A). Interestingly, the number of colonies of mixed cell type, a subtype of colonies that formed from an even earlier stage of stem/progenitor cells, tended to decrease in the Ovx group compared with the C group (19.5 ± 4.6 *vs.* 26.7 ± 3.5, *P *= 0.09; Fig. 4B), but the decrease was completely recovered by estrogen replacement treatment (30.2 ± 5.1, *P *= 0.57 *vs.* C group; Fig. 4B).

### Estrogen deficiency increased the number of CD105^+^ mesenchymal stem cells in the bone marrow

**Figure 5:**
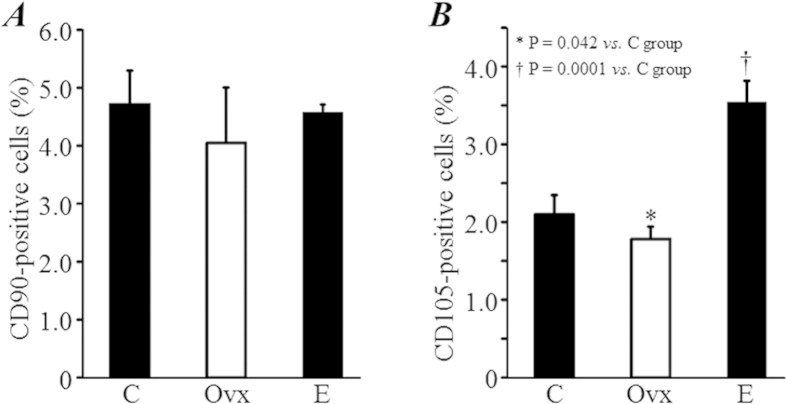
The number of mesenchymal stem cells. The expression of CD90 (**A**) and CD105 (**B**), two markers for mesenchymal stem cells in bone marrow mononuclear cells, were measured by flow cytometry.

We also measured the expressions of CD90 and CD105, two popular markers used for the identifying mesenchymal stem cells. The expression of CD90 in BM-MNCs did not significantly differ among groups (Fig. 5A). However, the expression of CD105 in BM-MNCs was significantly lower in the Ovx group compared with the C group (1.78 ± 0.25% *vs.* 2.10 ± 0.16%, *P *= 0.042; Fig. 5B), and was at the highest level in the E group (3.53 ± 0.29%, *P *= 0.0001 *vs.* C group; Fig. 5B).

### Estrogen deficiency did not change the number of satellite cells in skeletal muscle

**Figure 6:**
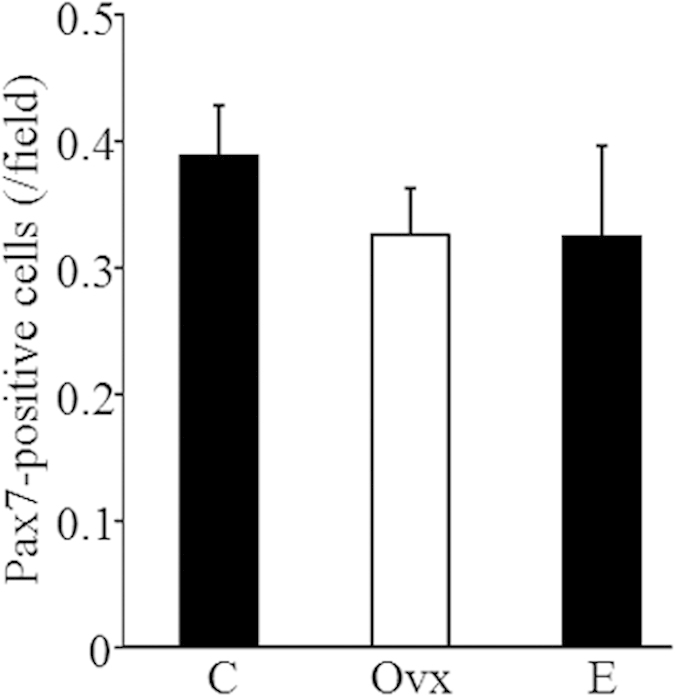
The number of satellite cells. Satellite cells were detected in tibialis anterior muscles by immunostaining with an anti-Pax7 antibody, and the Pax7-positive cells in randomly selected fields was counted under fluorescence microscopy.

By counting the number of Pax7^+^ satellite cells within the tibialis, we found that there was no significant difference among groups (Fig. 6).

## Discussion

The present study was designed to examine the hypothesis that estrogen deficiency induces a decrease in the quantity and quality of tissue-specific stem cells, thereby contributing to postmenopausal secondary disorders in different tissues/organs. Using an ovariectomy model in young healthy female mice, we found that estrogen deficiency increased the number, but likely impaired the function, of hematopoietic stem/progenitor cells. Estrogen deficiency also significantly decreased the number of CD105^+^ mesenchymal stem cells in bone marrow, but did not change the number of Pax7^+^ satellite cells in skeletal muscles. Our data shows the heterogeneous effects of estrogen deficiency in different types of tissue-specific stem cells, suggesting a likely and direct relationship between the estrogen deficiency-induced impairment of stem cells and postmenopausal disorders.

Although estrogens are generally known as female reproductive hormones, the functions of estrogens in non-reproductive tissues, such as brain, bone, and cardiovascular systems, are well-defined from previous studies^13^,^14^,^15^. The biological activities of estrogens are mediated by two estrogen receptor (ER) isoforms, namely ERα and ERβ^15^. As stem cells in various tissues express ERs, we examined the role of estrogen in stem cells *in vivo*. We used the well-established ovariectomy-induced estrogen deficiency model in young healthy female mice, which were subsequently treated with estrogen replacement or placebo. Estrogen deficiency was confirmed by the presence of uterine hypotrophy following ovariectomy. Uterine size was significantly increased by subcutaneous insertion with a slow-release pellet of 17β-estradiol, an easy method popularly used for estrogen replacement. However, compared with healthy mice in the control group, the uterine weight was much lower in mice receiving estrogen replacement after ovariectomy. Although we did not monitor the levels of estrogen in mice, the small size of the uterus might be due to not enough estrogen being released from the slow-release pellet to maintain it at physiological levels, especially during the later periods after subcutaneous insertion.

A recent study found that the treatment with 17β-estradiol enhances the cell cycle and increases the numbers of LSK (lin-/Sca-1+/c-kit+) hematopoietic stem cells in wild-type healthy mice, but not in ERα-knockout mice^8^. Unexpectedly, our data revealed that bone marrow mononuclear cells from ovariectomized mice contained more c-kit^+^ hematopoietic stem/progenitor cells, but showed an impaired capacity of growth for colonies of mixed cell type *in vitro*. Estrogen replacement treatment increased c-kit^+^ hematopoietic stem/progenitor cells to an even higher level, and also improved the colony forming capacity. Similar to our data, a very recently published study has also observed an increased number of hematopoietic stem cells with impaired function in aged mice^16^. It is quite possible that estrogen deficiency may also increase the number, but decrease the quality of hematopoietic stem/progenitor cells. However, further study with longer follow-up is required to answer whether estrogen deficiency decreases the number of hematopoietic stem cells. It is also highly required to understand the molecular mechanism on estrogen deficiency-induced increase of c-kit-positive stem/progenitor cells.

The expression of CD105, one of the most popularly used markers for the identifying mesenchymal stem cells, was significantly decreased in BM-MNCs from ovariectomized mice, but increased with estrogen replacement. This suggests that estrogen deficiency might inhibit mesenchymal stem cell function, as it did not change the expression of CD90, another marker of mesenchymal stem cells. It is possible that some mononuclear cells that are not the phenotype of mesenchymal stem cells, such as matured T cells, were positive for CD90, also known as Thy-1, which was originally identified on murine thymocytes and is expressed in T cells^17^. If this is possible, then estrogen deficiency may reduce the number of mesenchymal stem cells in bone marrow.

Satellite cells are the myogenic progenitor cells of postnatal skeletal muscles and reside at the periphery of myofibers in a quiescent state^18^. Controversial results have previously been reported on the role of estrogen in regulating satellite cells. It has been found that estrogen increases the number of satellite cells after exercise^19^, and selective activation of ERβ stimulates skeletal muscle growth and regeneration^11^. In contrast, estrogen has also been demonstrated to repress myogenic differentiation of satellite cells^20^. Although these results lack functional analysis, our data revealed that estrogen deficiency did not change the number of satellite cells within 2 months of follow-up. Long-term follow-up is needed to confirm this finding.

The reason for the heterogeneity of estrogen deficiency effects in different types of stem cells remains unclear. Many studies have reported anti-oxidative and anti-inflammatory effects of estrogens, which may be attributed to their phenolic hydroxyl group^21^. However, the phenolic hydroxyl group present at the C3 position of the A ring of estrogen can be oxidized, either by accepting an electron or by losing a proton^22^. In addition, estrogens also produce ROS by increasing mitochondrial activity, thus meaning that they also act as pro-oxidants. The controversial role of estrogens as pro-oxidants or anti-oxidants is largely dependent on cell type and different expression levels of ERs in a particular cell. Our data indicate that estrogen deficiency does not significantly change the intracellular ROS level and the expression of CXCR4 in BM-MNCs, suggesting a mineral role of estrogens as an anti-oxidant and anti-inflammatory.

It is critical to compare the expression levels of ERs in different cells after treatments. Unfortunately, we failed to quantify the expression of ERS by flow cytometry due to the quality problem of antibodies. Therefore, we tried to measure the mRNA expression of ERs in c-kit^+^ hematopoietic stem cells and satellite cells (Supplementary Figure). Interestingly, the expression of ERβ was higher in c-kit^+^ hematopoietic stem/progenitor cells than the matured c-kit^−^ mononuclear cells from bone marrow. However, the expressions of ERα and ERβ were even lower in the satellite cells compared with differentiated/matured muscle fibers. Although we did not measure the changes of ERs in these cells after different treatments, the effect of estrogen deficiency in stem cells may depend, at least partially, on the expression levels of ERs, which provides reasonable explanation on the heterogeneity of estrogen deficiency-induced changes in different types of stem cells.

In summary, the results of this study revealed that estrogen deficiency heterogeneously affects tissue-specific stem cells. Further study, including a longer follow-up, is required to ascertain the estrogen deficiency-induced impair of tissue-specific stem cells. This may finally uncover the relevant molecular and cellular mechanisms, and provide novel strategy for the prevention and treatment of postmenopausal disorders.

## Materials and Methods

### Animals

Six-week-old female C57BL/6 mice were used in this study. All animals were purchased from Charles River Laboratories, Kanagawa Japan. All experiments were approved by the Institutional Animal Care and Use Committee of Nagasaki University (No. 120721004), and experiments were performed in accordance with the institutional and national guidelines.

### Ovariectomy and estrogen replacement treatment

Mice were ovariectomized under anesthesia, and then randomly received subcutaneous insertion of a placebo pellet (Ovx group, n = 8), or a slow-release 17β-estradiol pellet (0.01 mg/60 days, Innovative Research of America, FL USA) for estrogen replacement (E group, n = 7), as previously described^23^. Sham-operated mice were used as the healthy control (C group, n = 8). All mice were killed 2 months after treatments. The body weight and uterine weight were measured, and other tissue samplings and assessments were performed as described in the following sections.

### Collection of mononuclear cells from bone marrow

Bone marrow cells were collected from the femur and tibia. Bone marrow mononuclear cells (BM-MNCs) were isolated by density gradient centrifugation^24^,^25^, and then counted using a Nucleo Counter cell-counting device (Chemotetec A/S, Denmark).

### Flow cytometry

To measure the number of c-kit-positive (c-kit^+^) stem/progenitor cells in the freshly isolated BM-MNCs, we labeled cells with a PE-conjugated anti-mouse c-kit antibody (eBioscience, CA USA) for 30 min. Respective isotype controls were used as a negative control. After washing, quantitative flow cytometry analysis was performed using a FACSCalibur instrument (Becton Dickinson, USA)^24^,^25^. The acquired data was analyzed using Cell Quest software (Becton Dickinson).

BM-MNCs were also stained with goat and rat monoclonal antibodies against CD90 (Abcam, UK), CD105 (Abcam), CXCR4 (R&D Systems, USA), followed by their respective FITC-conjugated secondary antibodies. The expressions of CD90, CD105, and CXCR4 in BM-MNCs were analyzed by flow cytometry as described above.

### Detection of intracellular levels of reactive oxygen species (ROS)

To measure the intracellular levels of ROS, freshly isolated BM-MNCs were incubated with 10 μM CM-H_2_DCFDA (Molecular Probes Inc., OR USA), at 37 °C for 30 min^26^. After washing, the fluorescence intensity in cells was measured by flow cytometry as described above.

### Colony-forming assay

The colony-forming capacity of the isolated BM-MNCs was estimated using mouse methylcellulose complete medium^27^, according to the manufacturer’s instructions (R&D System, MN USA). Briefly, 2 × 10^4^ cells were mixed well with 1 ml of medium, plated in 3-cm culture dishes, and incubated at 37 °C in a 5% CO_2_ incubator. The formation of colonies was observed under a microscope, and the total number of colonies in each dish was counted after 7 days of incubation. We also counted the number of colonies of mixed cell type, in which at least two different types of cell were growing from a single stem/progenitor cell. The mean number of colonies in duplicate assays was used for statistical analysis.

### Immunohistological staining

To detect the presence of satellite cells, tibialis anterior muscles were dissected, frozen in liquid nitrogen, and stored in a −80 °C refrigerator. Muscle tissues were sectioned into 5-μm-thick sections and fixed with 4% paraformaldehyde for 10 min. After blocking with DAKO Protein Block (DAKO, Denmark), sections were incubated with anti-laminin α2 antibody (Alexis, CA, USA) to stain the basal membrane of muscle fibers. Satellite cells that localized to the inside of the skeletal basal membrane of muscle fibers were stained using an anti-Pax7 antibody (Santa Cruz, CA, USA). All immunostained samples were visualized using their respective secondary antibodies conjugated with Alexa Fluor 488 or 568 (Life Technologies, Tokyo, Japan). The nuclei were labeled with DAPI. We counted the number of Pax7-positive satellite cells directly under a fluorescence microscope with 200-fold magnification. We randomly selected at least 10 fields from three sections of each animal, and the average cell count per field was used for statistical analysis.

### Statistical analyses

All results are presented as the means ± standard error mean (SEM). The statistical significance was determined by one-way analysis of variance followed by the Bonferroni post hoc test (Dr. SPSS II, Chicago, IL). Differences were considered significant when *P *< 0.05.

## Additional Information

**How to cite this article**: Kitajima, Y. *et al.* Estrogen deficiency heterogeneously affects tissue specific stem cells in mice. *Sci. Rep.* **5**, 12861; doi: 10.1038/srep12861 (2015).

## Supplementary Material

Supplementary Information
