# Supplementary material for: Estrogen deficiency heterogeneously affects tissue specific stem cells in mice
Source: Sci Rep. 2015 Aug 6;5:12861. doi: 10.1038/srep12861 (PMC4526849; doi:10.1038/srep12861)

Estrogen deficiency heterogeneously affects tissue specific stem cells in mice

Yuriko Kitajima, Hanako Doi, Yusuke Ono, Yoshishige Urata, Shinji Goto, Michio Kitajima, Kiyonori Miura, Tao-Sheng Li, Hideaki Masuzaki

Supplementary Figure.

A) The mRNA expression of ERβ in c-kit+ hematopoietic stem/progenitor cells and the matured c-kit-negative mononuclear cells from bone marrow.
B) The mRNA expressions of ERα and ERβ in the satellite cells after 3 days culture in growth medium and differentiated medium.

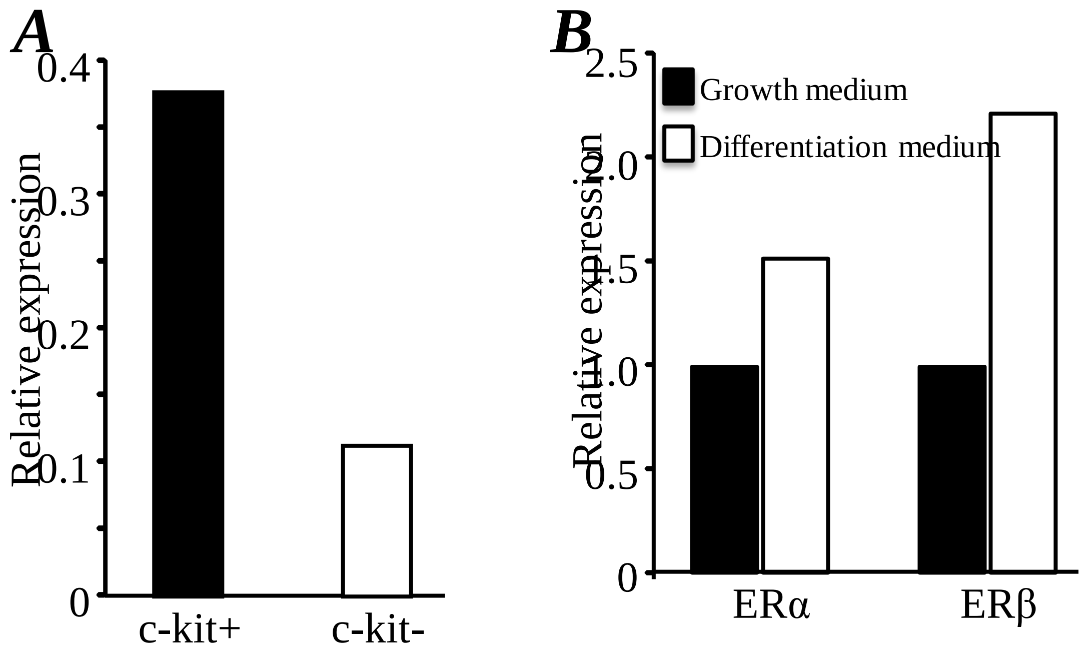

Supplement: Supplementary Information [file srep12861-s1.pdf]
